# Novel T-cell subsets as non-invasive biomarkers of vascular damage along the predialysis stages of chronic kidney disease

**DOI:** 10.3389/fmed.2024.1460021

**Published:** 2024-12-09

**Authors:** Julia Martín-Vírgala, Daniel Miranda-Prieto, Sara Fernández-Villabrille, Beatriz Martín-Carro, Nerea González-García, Joaquín Bande-Fernández, Carmen Díaz-Corte, José Luis Fernández-Martín, Cristina Alonso-Montes, Ana Suárez, Sara Panizo, Manuel Naves-Díaz, Javier Rodríguez-Carrio, Natalia Carrillo-López

**Affiliations:** ^1^Metabolismo Óseo, Vascular y Enfermedades Inflamatorias Crónicas, Instituto de Investigación Sanitaria del Principado de Asturias (ISPA), Oviedo, Spain; ^2^Bone and Mineral Research Unit, Hospital Universitario Central de Asturias, Oviedo, Spain; ^3^Resultados en Salud 2040 (RICORS2040, Kidney Disease), Oviedo, Spain; ^4^Area of Immunology, Department of Functional Biology, Instituto de Investigación Sanitaria del Principado de Asturias (ISPA), University of Oviedo, Oviedo, Spain; ^5^Investigación básica y traslacional en enfermedades inflamatorias crónicas, Instituto de Investigación del Principado de Asturias (ISPA), Oviedo, Spain; ^6^Nephrology Unit, Hospital Universitario Central de Asturias, Oviedo, Spain; ^7^Departamento de Medicina, Universidad de Oviedo, Oviedo, Spain

**Keywords:** aortic stiffness, non-invasive, CKD, inflammation, Tang cells, vascular indices

## Abstract

**Introduction:**

Cardiovascular disease is the major cause of premature death in chronic kidney disease (CKD) and vascular damage is often detected belatedly, usually evaluated by expensive and invasive techniques. CKD involves specific risk factors that lead to vascular calcification and atherosclerosis, where inflammation plays a critical role. However, there are few inflammation-related markers to predict vascular damage in CKD. This study aimed to investigate immune populations in pre-dialysis patients to (i) identify subset alterations, (ii) assess longitudinal changes, and (iii) evaluate their applicability as biomarkers of subclinical vascular indices.

**Methods:**

43 pre-dialysis CKD patients in stages CKD-2 to CKD-5 and 38 controls were recruited at baseline and after 18-month follow-up. Aortic stiffness was determined by carotid-femoral pulse wave velocity (PWV) and abdominal aortic calcification was quantified by the Kauppila index on X-rays. Carotid intima-media thickness, the number of carotid plaques and adventitial neovascularization were evaluated by Superb Microvascular Imaging. Peripheral blood mononuclear cells were isolated and immune cell populations were assessed by flow cytometry: senescent T cells (CD4^+^CD28^null^), Tang (CD3^+^CD31^+^CD184^+^) and derived subsets, and monocyte subsets (classical, intermediate and non-classical; and ACE expression).

**Results:**

Senescent T cells were increased in CKD. Despite Tang levels were unchanged compared to controls, this subset exhibited enhanced immunosenescence traits (CD28^null^ and inverted CD4^+^CD8^+^ ratio) in CKD. Furthermore, Tang were negatively correlated with CKD progression. Slight alterations within monocyte subsets were observed. These findings were validated at the 18-month follow-up. Tang were correlated with several subclinical indices, and further analyses revealed an independent effect on PWV and their potential value as biomarkers. Intermediate monocytes were positively correlated with PWV.

**Conclusion:**

Pre-dialysis CKD stages are hallmarked by alterations in immune cell populations related to vascular homeostasis, including early T-cell immunosenescence traits and a stage-dependent Tang depletion, which was independently related to vascular stiffness. All these features were replicated upon follow-up, thus providing validation toward our results. Our findings pave the ground for future studies addressing the functional contribution of these cellular mediators at the local level, assessing their potential predictive value in the long-term and implementing preventive strategies in the clinical setting.

## Introduction

Cardiovascular (CV) disease is the major cause of premature death in chronic kidney disease (CKD), being a critical concern for clinical management. Furthermore, dialysis treatment highly exacerbates CV risk (1). Therefore, detecting and predicting cardiovascular impairment in earlier stages of CKD holds great clinical potential.

Currently, vascular damage in CKD is often detected belatedly, and it is usually evaluated using expensive and invasive techniques (2). However, some non-invasive tools might be useful to assess vascular damage in CKD, such as pulse wave velocity (PWV) -the gold-standard technique to assess aortic stiffness- or, more recently proposed, Superb Microvascular Imaging (SMI) ultrasound to measure adventitial neovascularization (3). Of note, need of trained experts and limited availability in some areas pose important challenges for accessibility and cost-effectiveness.

Apart from the traditional CV risk factors, the CKD setting involves specific risk factors which lead to vascular calcification and atherosclerosis, such as bone and mineral metabolism alterations, dyslipidemia or systemic inflammation (4). Immunosenescent cells display an inflammatory profile, as they secrete proinflammatory cytokines, infiltrate tissues and escape apoptosis and immune regulation (5). As this inflammatory state involves macrophage activation and endothelial cell killing, immunosenescence has been linked to CV disease (6, 7), although its exact origin in CKD is ill-defined.

Monocytes also play a pivotal role in atherosclerosis (8). Recently, the relevance of monocyte heterogeneity has emerged (9), and specific monocyte subpopulations have been reported to be increased in CKD, being also predictors of CV disease in CKD (10–12). However, little is known about their possible association with subclinical vascular indices in CKD patients, especially in non-dialysis populations.

Furthermore, the role of vascular protective mechanisms has gained relevance along last decade by virtue of their ability to maintain vascular homeostasis. Angiogenic T-cells (Tang) are a novel T-cell subset involved in vasculogenesis and vascular repair (12). Tang cells have been described to be impaired in a number of inflammatory and vascular diseases (13, 14). However, the potential role of Tang in CKD has been barely studied (11) and only limited to end-stage renal disease.

Preliminary results from our group provided novel insight about the use of PWV and SMI to evaluate vascular damage and its progression in CKD (3). We found increased PWV in advanced vascular stiffness and reported, for the first time, increased adventitial neovascularization in these patients. Taken together, these findings support the use of PWV and SMI over other imaging approaches and, more importantly, reinforced their value for patient monitoring, paving the way for preventive CV strategies. Nevertheless, it would be of great relevance to deepen into the connection with inflammatory circuits.

Gaining understanding toward these processes may not only help in the identification of potential disease targets and pathogenic mechanisms, but also to inform clinically relevant biomarkers to predict vascular damage in CKD. However, evidence is limited, especially in pre-dialysis populations. Therefore, based on the data about vascular damage from the above-mentioned work, this study aimed to investigate immune cell populations in patients from stages CKD-2 to CKD-5 previous to dialysis in order to (i) identify subset alterations, (ii) assess longitudinal changes, and (iii) evaluate their applicability as biomarkers of subclinical vascular indices, such as cIMT, number of carotid plaques, PWV, adventitial neovascularization and aortic calcification.

## Methods

### Study participants

The study was performed in 43 CKD patients (25 men and 18 women) that belonged to the Nephrology Unit (Hospital Universitario Central de Asturias). Sample size calculations were performed *a priori* based on previous data on PWV values between patients and controls (11), with an alfa = 0.050 and a power = 0.90. CKD patients were divided into four groups according to their estimated glomerular filtration rate (eGFR) and following the 2017 KDIGO clinical guidelines (15): CKD-2/3a (45–89 mL/min/1.73 m^2^), CKD-3b (30–44 mL/min/1.73 m^2^), CKD-4 (15–29 mL/min/1.73 m^2^) and CKD-5 (<15 mL/min/1.73 m^2^). A group of 38 individuals was recruited as a healthy control (HC) group (18 men and 20 women). Control subjects were recruited among individuals from the same geographical area attending the blood sample collection area and were asked if they would consent to participate in the study. They were recruited to ensure a similar age and men/women ratio as the CKD group. The analysis was carried out at baseline and after an 18-month follow-up.

The exclusion criteria for patient and control populations were (a) diabetes mellitus, (b) abdominal aneurism or intermittent claudication, (c) previous carotid surgery (d) concomitant immune-mediated disease or cancer diagnosis, (e) ongoing immunosuppressive treatment, (f) recent or current infection or (g) pregnancy.

The following clinical and anthropometric data were collected: age, sex, body mass index (BMI), arterial pressure, heart rate, pharmacological treatments and biochemical parameters (Table 1). Creatinine, calcium and phosphorus were determined using a Cobas 702 equipment (Roche Diagnostics), PTH was determined using an electrochemiluminescence immunoassay (ECLIA, Roche Diagnostics) and intact FGF23 was determined with a chemiluminescence immunoassay (CLIA, DiaSorin).

**Table 1:** Demographic, laboratory, and clinical parameters of study participants at baseline.

	HC (*n* = 38)	CKD (*n* = 43)	*p*-value
Demographics			
Age (years)	66.5 ± 4.6	66.7 ± 8.6	0.920
Sex (*n* women/men)	20/18	18/25	0.456
Clinical features			
BMI (kg/m^2^)	27.2 ± 4.6	28.9 ± 5.0	0.120
Systolic blood pressure (mm Hg)	132.0 [22.0]	140.0 [27.0]	0.008
Diastolic blood pressure (mm Hg)	75.5 [12.0]	79.5 [19.3]	0.093
Heart rate (beats per min)	62 ± 7	67 ± 14	0.028
eGFR (mL/min/1.73 m^2^)	81.5 [12.8]	32.0 [24.5]	<0.001
Creatinine (mg/dL)	0.83 [0.26]	1.91 [1.52]	<0.001
Calcium (mg/dL)	9.53 ± 0.27	9.53 ± 0.44	0.924
Phosphorus (mg/dL)	3.64 [0.46]	3.44 [0.76]	0.091
PTH (pg/mL)	52.0 [19.0]	103.0 [86.0]	<0.001
FGF23 (pg/mL)	55.19 [18.36]	127.5 [125.99]	<0.001
Calcidiol (ng/mL)	28.97 ± 13.25	26.70 ± 11.38	0.413
Traditional risk factors, *n* (%)			
Hypertension	6 (15.8)	35 (81.4)	<0.001
Dyslipemia	10 (26.3)	31 (72.1)	<0.001
Treatments, *n* (%)			
Anti-hypertensive drugs (any)	7 (18.4)	34 (79.1)	<0.001
ACEi	4 (10.5)	23 (53.5)	<0.001
Diuretics	4 (10.5)	18 (41.9)	0.004
Statins	5 (13.2)	34 (79.1)	<0.001
Paracalcitol	1 (2.6)	9 (20.9)	0.031
Calcimimetics	0 (0)	2 (4.7)	0.530

Variables were summarized as mean ± SD, median [interquartile range] or *n* (%). Differences were assessed by chi-square tests, Student t tests or Mann-Withney U tests, as appropriate. HC, healthy controls; CKD, chronic kidney disease; BMI, body mass index; eGFR, estimated glomerular filtration rate; PTH, parathyroid hormone; FGF23, fibroblast growth factor 23; ACEi, angiotensin converting enzyme inhibitors.

### Subclinical vascular indices

Aortic stiffness was assessed in the right side by carotid-femoral pulse wave velocity (PWV), using the Complior Analyze equipment (ALAM Medical). Values above 90% quality were considered, and results are the average of three optimal measurements. A PWV value above 10 m/s was considered as indicative of vascular stiffness, according to guidelines (16).

B-mode ultrasound, using the probe Superb Microvascular Imaging (SMI) Ultrasound (Toshiba Aplio 500), was performed with a Toshiba-Aplio XG equipment (Toshiba American Medical Systems) to analyze: left carotid intima-media thickness (cIMT), number of carotid plaques (either cIMT <1.5 mm or a focal thickening going over into the arterial lumen by at least 50% of the surrounding cIMT value), if the plaques were calcified, and adventitial neovascularization in the carotid and femoral arteries. Image J software was used to quantify the number of neovasa and area of adventitial neovascularization.

Aortic calcification was assessed on a lateral radiograph by the lumbar vertebrae (L1 to L4). Vascular calcification was quantified by the semi-quantitative Kauppila index, using the following score: 0, no calcification; 1, 1/3 of the vertebral body length was calcified; 2, 2/3 of the vertebral body length was calcified and 3, the whole length of the vertebral body was calcified (17).

All vascular measurements were performed by operators blinded to the study participants.

### Assessment of immune cell populations

Blood samples were collected in EDTA-containing tubes, and these were immediately processed for peripheral blood mononuclear cells (PBMCs) isolation by centrifugation on density gradient using Ficoll–Hypaque (Biowest, Belgium) according to conventional protocols (11).

Immune cell populations were identified based on their extracellular markers by flow cytometry as previously described (11). Specific fluorochrome panels were designed and panel-specific fluorescence compensation settings were carried out. In brief, PBMCs were incubated with CD14-FITC (Immunostep, Spain), CD16-APC-Cy7 (BioLegend, Germany) and angiotensisn converting enzyme (ACE) ACE-APC (Miltenyi Biotech), or CD3-PerCP-Cy-5,5 (Tonbo Biosciences, Belgium), CD184-PE-Cy7 (BD Biosciences, Germany), CD31-FITC (BD Biosciences), CD4-PE (Immunostep) and CD28 APC-Cy7 (Thermo Fisher, Germany), or corresponding isotype antibodies for 30 min at 4°C protected from light. Then, stained cells were washed with PBS and analyzed in a FACS Canto II (BD Biosciences) with FACS Diva 6.5 software. Furthermore, regular testing for spectral overlap was performed with selected pairs of fluorochromes on a regular basis to ensure traceability and instrument stability.

Lymphocytes and monocytes populations were defined according to their FSC/SSC signal and gating strategies were follow as previously reported (11) for the identification of Tang (CD3^+^CD31^+^CD184^+^), Tang subsets (CD4^+^Tang and CD8^+^Tang), senescent T-cells (CD4^+^CD28^null^), monocyte subsets [classical (CD14^+^CD16^−^)], intermediate (CD14^+^CD16^+^) and non-classical (CD14^low^CD16^+^) monocytes and ACE expression. The frequency of each population was referred to as a percentage of the immediate parental gate/population, unless otherwise stated.

### Statistical analysis

Results are shown as median [interquartile range] or mean ± standard deviation according to data distribution. Categorical variables were summarized as the absolute number (*n*) and percentage (%) within the whole group. Statistical comparisons between groups were performed using Mann–Whitney test (non-parametric analysis), t test (parametric analysis), or chi-squared test (categorical variables). Correlations were assessed by Spearman’s rank test. Hedge’s g statistic was calculated for each reported significant difference in cellular populations in our study (18), with values of *g* > 0.5 and *g* > 0.8 considered of medium and large effect, respectively, as previously described (19). Logistic and linear regression models, either univariate or multivariate adjusted by confounders (either continuous or categorical variables), were used to evaluate the associations between immune cell populations and subclinical vascular indices. Odds ratios (OR) and 95% confidence intervals (CI) were computed. Multivariate models were built in a two-step process after observing significant associations (*p* < 0.100) in the univariate analyses. Area under the curve (AUC) for ROC analysis and classification statistics were used to evaluate the role of cell subsets as biomarkers. A *p* value lower than 0.050 was considered statistically significant. Statistical analyses were performed under SPSS 27.0 and GraphPad Prism 8.0 for Windows.

## Results

### Demographics, clinical features, traditional risk factors and treatments

A total of 43 CKD patients and 38 HC were recruited for this study. Table 1 shows, at baseline, demographics, clinical features, traditional risk factors and pharmacological treatments in CKD-2/3a to CKD-5 patients and the control group. No differences were found in age and sex between the control group and CKD patients (Table 1). Complete blood counts revealed no differences between controls and CKD patients for lymphocytes (2.02 [0.75] vs. 1.80 [1.09] 10^3^ cells/μL, *p* = 0.089) or monocytes (0.500 [0.18] vs. 0.53 [0.27]·103 cells/μL, *p* = 0.484) subsets. A total number of 8 patients were lost in the follow-up due to cancer diagnosis, renal replacement therapy or drop out.

In CKD patients, no statistical differences were found between the baseline study and the 18-month follow-up in any clinical features (Supplementary Table S1), except for increased serum levels of creatinine (*p* = 0.002) and FGF23 (*p* = 0.010).

### Analysis of T-cell subsets

**Figure 1:**
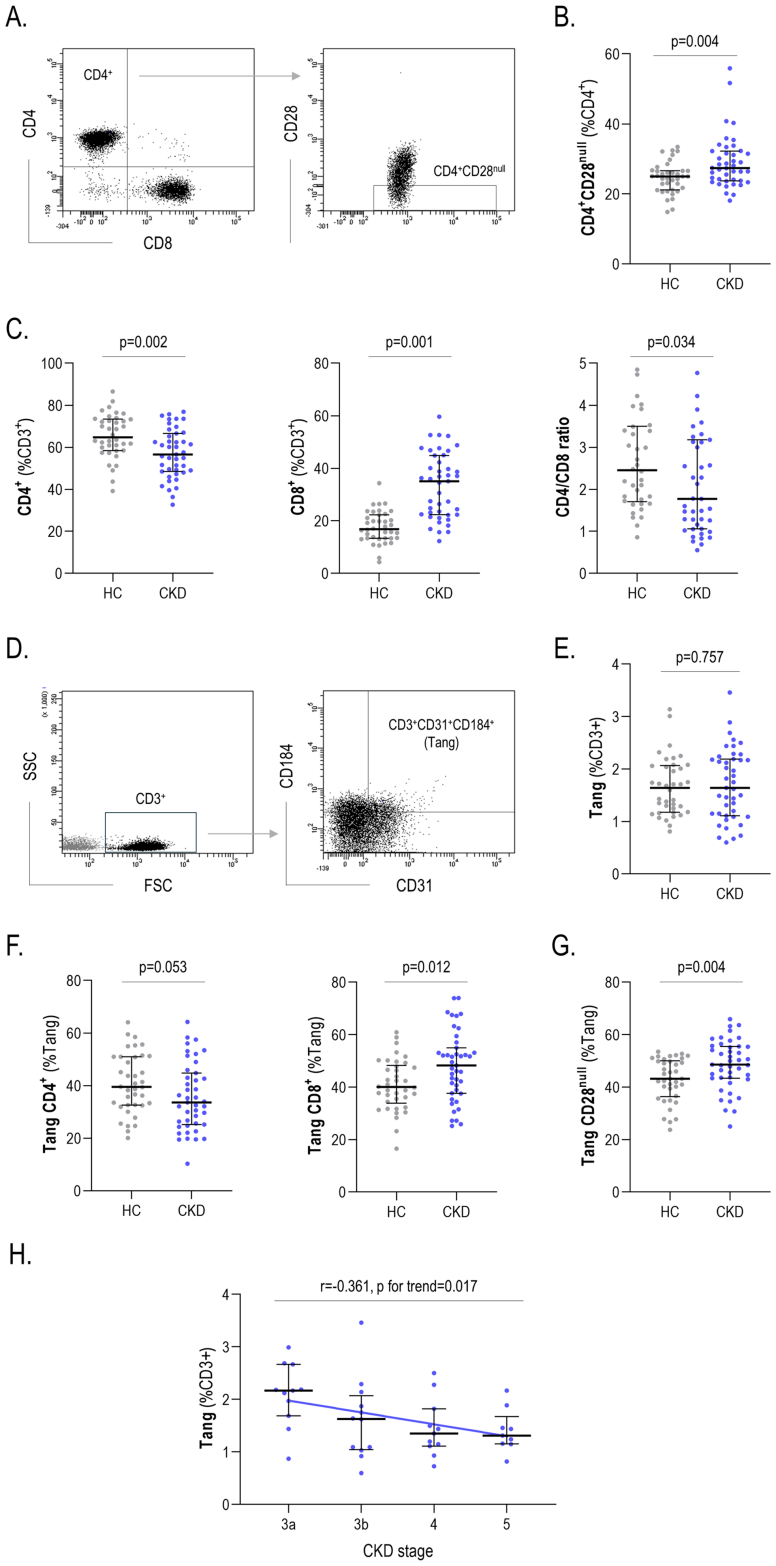
Analysis of T-cell subsets. CD4^+^CD28^null^ cells quantification by flow cytometry **(A)**, and their levels were compared between HC and CKD (*g* = 0.78) **(B)**. Equivalent analyses were performed with CD4^+^ (*g* = 0.61) and CD8^+^ (*g* = 0.75) subsets and CD4/CD8 ratio (*g* = 0.48) **(C)**. Tang frequency was also assessed by flow cytometry **(D)**. Tang levels were compared between HC and CKD **(E)**, and equivalent analyses were performed with CD4^+^Tang (*g* = 0.54), CD8^+^Tang (*g* = 0.70) **(F)**, and CD28^null^Tang subsets (*g* = 0.75) **(G)** The levels of Tang were also analyzed according to CKD stages **(H)**. Differences were assessed by Mann-Withney U or correlation tests. Scatter plots depict the distribution of individual values, and each dot corresponds to one individual: HC (gray), CKD (blue). Upper, medium and lower bars represent 75th, 50th (median) and 25th percentiles. CKD, chronic kidney disease; HC, healthy control.

Immunosenescent T-cells (CD4^+^CD28^null^) were evaluated by flow cytometry (Figure 1A), and a marked increase was observed in CKD patients compared to HC out of CD4^+^ cells (Figure 1B). Equivalent results were obtained within the total lymphocyte population (6.15 [1.11] vs. 3.50 [0.85] %, *p* = 0.025) and when absolute numbers were computed (*p* < 0.050). CKD patients exhibited an expansion of the CD8^+^ population at the expense of the CD4^+^ subset, thus presenting with a strong decrease in the CD4/CD8 ratio compared with the HC group (Figure 1C).

Next, the Tang population was also assessed by flow cytometry (Figure 1D). Although no differences were observed regarding the total numbers of Tang both relative to the CD3^+^ (Figure 1E), to the lymphocyte gates (HC: 1.03 [0.55] vs. CKD: 0.87 [0.54]%, *p* = 0.088) or when absolute counts were considered (*p* = 0.078), differences were observed within this population. First, the frequency of CD4^+^Tang and CD8^+^Tang subsets were altered between groups (Figure 1F). Second, CD28^null^Tang cells were strongly elevated in CKD compared to their HC counterparts (Figure 1G).

Interestingly, Tang levels showed a progressive decline across CKD subsets (*r* = −0.361, *p* for trend = 0.017) (Figure 1H). Similar results were retrieved when absolute levels were analyzed (*r* = −0.429, *p* = 004). No associations were observed with the rest of subsets (all *p* > 0.050). No associations were observed with demographics, biochemical parameters, or C reactive protein (CRP) levels (all *p* > 0.050). Furthermore, no associations with traditional CV risk factors or medications (Supplementary Table S2) were observed.

Taken together, all these findings revealed changes in several T-cell subsets which suggest a profound immunosenescence within this compartment along the whole CKD spectrum.

### Analysis of monocyte subsets

**Figure 2:**
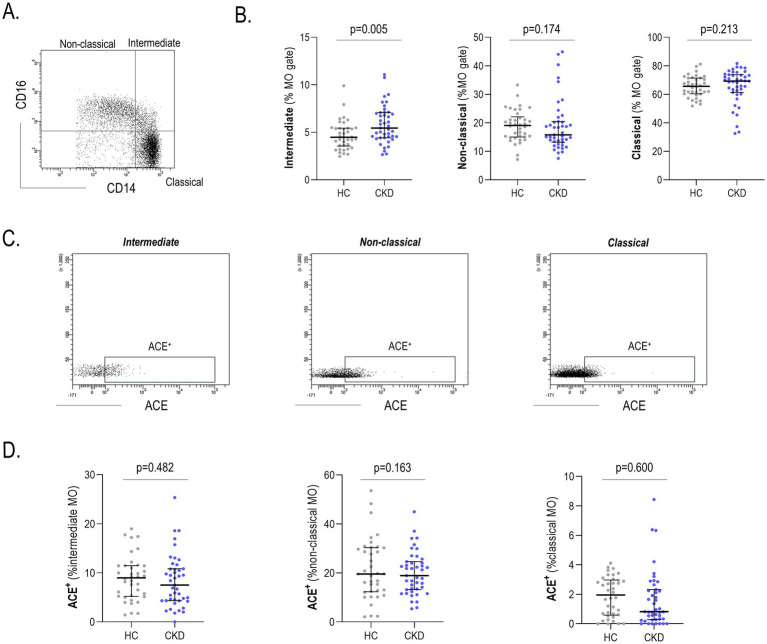
Analysis of monocyte subsets. Monocyte subsets were identified based on their CD14/CD16 expression **(A)**, and their levels were compared between HC and CKD (intermediate: *g* = 0.65) **(B)**. Equivalent analyses were performed with the ACE expression **(C)** for each subset **(D)**. Differences were assessed by Mann-Withney U. Scatter plots depict the distribution of individual values, and each dot corresponds to one individual: HC (gray), CKD (blue). Upper, medium and lower bars represent 75th, 50th (median) and 25th percentiles. CKD, chronic kidney disease; HC, healthy control.

Monocytes were identified according their FSC/SSC properties within the PBMC fraction, and their subsets were assessed based on their CD14/CD16 differential expression (Figure 2A). The CKD group was hallmarked by a slight increase in the frequency of intermediate monocytes (CD14^+^CD16^+^), whereas no differences were observed for their classical (CD14^+^CD16^−^) and non-classical (CD14^low^CD16^+^) counterparts (Figure 2B). Equivalent results were retrieved when frequencies were computed out of total PBMCs (intermediate: *p* = 0.010, classical: *p* = 0.205, and non-classical: *p* = 0.427) and when absolute counts were analyzed (*p* = 0.012, *p* = 0.342 and *p* = 0.540, respectively). Furthermore, the expression of ACE was assessed on each monocyte subset (Figure 2C). Of note, ACE expression on monocyte subsets did not differ between CKD and HC groups (Figure 2D).

Neither monocyte subsets nor ACE expression were associated with demographics, biochemical parameters or CKD stages (all *p* > 0.050). Similarly, no associations with traditional CV risk factors or medications (Supplementary Table S3) were found.

All these results suggest that CKD patients exhibited a mild alteration within the monocyte pool, unrelated to disease stages or clinical features.

### Longitudinal changes in immune cell populations

Next, whether fluctuations in these populations occurred was tested along an 18-month follow-up in both CKD and HC groups.

**Figure 3:**
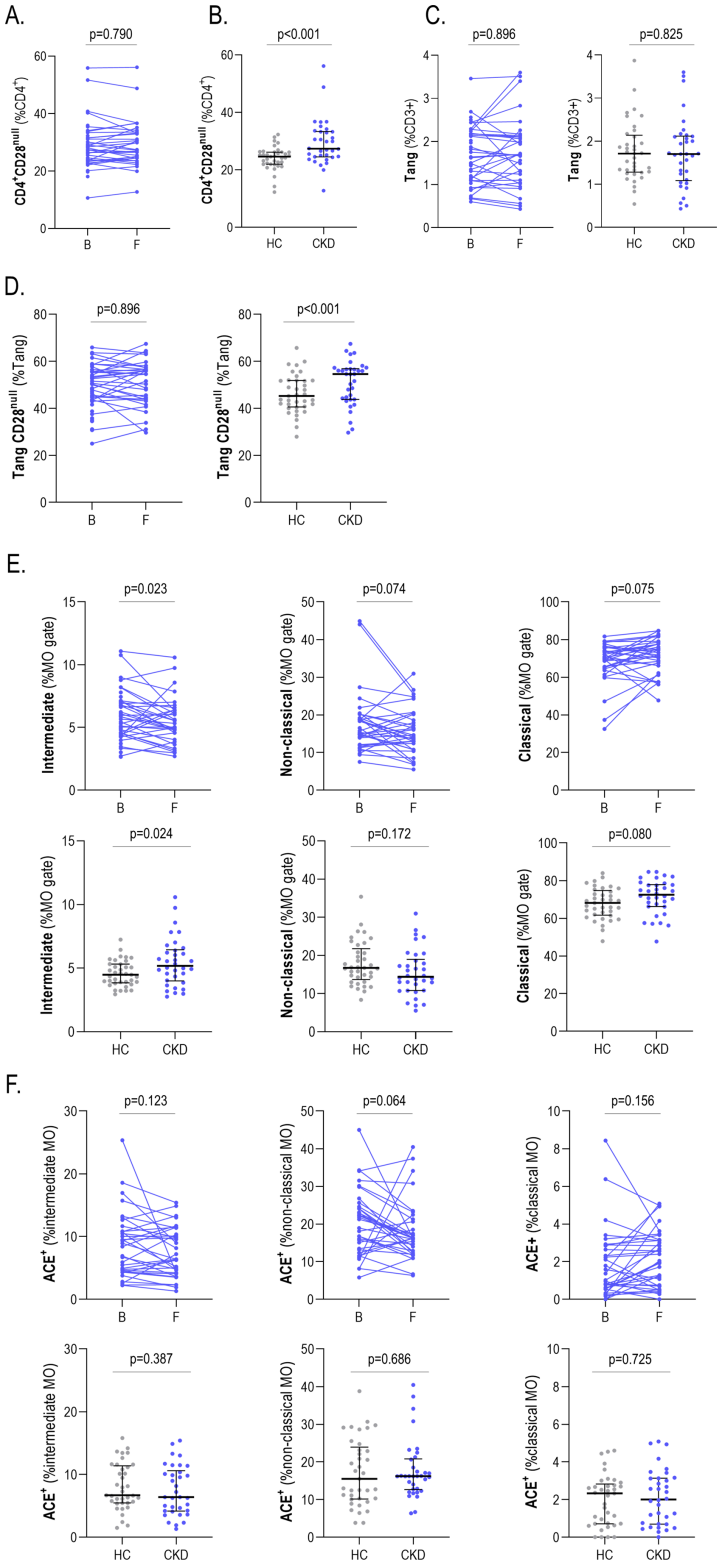
Analysis of longitudinal changes of immune cell populations upon follow-up. **(A)** The frequency of CD4^+^CD28^null^ cells was compared between baseline [B] and 18-month follow-up [F] samples in CKD patients. Differences between HC and CKD at 18-months follow-up were also compared (*g* = 0.69) **(B)**. Equivalent analyses were performed with Tang **(C)**, TangCD28^null^ (*g* = 0.72) **(D)**, monocyte subsets (intermediate: *g* = 0.60) **(E)** and ACE expression **(F)**. Differences were assessed by Wilcoxon paired pr Mann-Withney U tests, as appropriate. Scatter plots depict the distribution of individual values, and each dot corresponds to one individual: HC (gray), CKD (blue). Upper, medium and lower bars represent 75th, 50th (median) and 25th percentiles. B, baseline; CKD, chronic kidney disease; F, follow-up; HC, healthy control.

No changes in CD4^+^CD28^null^ frequency were registered at 18 months compared to baseline levels in CKD (Figure 3A). In fact, differences between groups remained as observed at baseline (Figure 3B). Equivalent figures were observed for Tang and TangCD28^null^ subsets (Figures 3C,D). When monocytes were analyzed, a similar picture was found, with no changes in any of the populations studied (Figures 3E,F). No changes in absolute levels for any cell population were registered (all *p* > 0.050).

Regarding clinical features, a total of 10 patients experienced progression (defined as a change to a higher CKD stage) at 18 months. CKD progression was not associated with differences in immune cell populations at baseline (Supplementary Table S4). Moreover, stratifying CKD patients according to progression status did not lead to different immune cell trajectories along follow-up (*p* > 0.050 in all cases).

Taken together, all these results suggest that changes in immune cell populations across the CKD spectrum are stable, at least in the absence of clinical progression.

### Association with subclinical vascular indices

Next, subclinical vascular indices, including vascular function/stiffness (PWV), atherosclerosis endpoints (cIMT, plaque occurrence and calcification), adventitial vascularization and vascular calcification (Kauppila index), were measured in CKD patients and HC both at baseline as well as after 18-months follow-up.

In order to evaluate whether altered immune cell populations could be linked to these subclinical vascular indices, an exploratory correlation analysis performed (Table 2). Interestingly, Tang levels were negatively associated with vascular stiffness, as well as with cIMT and carotid adventitial vascularization (Table 2), whereas no associations were found in the femoral territory. Although no associations with plaque presence were retrieved (Table 2), patients presenting plaque calcification (*n* = 17) exhibited lower Tang counts than their uncalcified-plaque counterparts (Supplementary Figure S1). Furthermore, the frequency of intermediate monocytes also paralleled vascular stiffness (Table 2). Of note, these correlations mirrored those observed with the 18-months assessments (Supplementary Table S4).

**Table 2:** Analysis of the associations between immune cell subsets and subclinical vascular indices at baseline.

	Number of carotid neovasa	Area of carotid neovasa (mm^2^/mm %)	Number of femoral neovasa (mm^2^/mm %)	Area of femoral neovasa	PWV m/s	cIMT mm	Carotid plaque (Yes/Not)	Femoral plaque (Yes/Not)
T-cell subsets								
Tang (% CD3^+^)	***r* = −0.378** ***p* = 0.012**	***r* = −0.406** ***p* = 0.007**	*r* = −0.261 *p* = 0.091	*r* = −0.263 *p* = 0.088	***r* = −0.598** ***p* < 0.001**	***r* = −0.363** ***p* = 0.017**	*p* = 0.210	*p* = 0.093
TangCD28^null^ (%Tang)	*r* = −0.114 *p* = 0.468	*r* = −0.078 *p* = 0.618	*r* = −0.256 *p* = 0.097	*r* = −0.259 *p* = 0.093	*r* = −0.029 *p* = 0.859	*r* = −0.047 *p* = 0.763	*p* = 0.339	*p* = 0.778
CD4^+^CD28^null^ (% CD4^+^)	*r* = −0.193 *p* = 0.215	*r* = −0.168 *p* = 0.282	*r* = 0.091 *p* = 0.562	*r* = 0.091 *p* = 0.560	*r* = −0.124 *p* = 0.433	r = −0.183 *p* = 0.242	*p* = 0.535	*p* = 0.452
Monocyte subsets								
Classical (% MO subset)	*r* = 0.053 *p* = 0.737	*r* = −0.012 *p* = 0.939	*r* = −0.180 *p* = 0.243	*r* = −0.182 *p* = 0.242	*r* = −0.046 *p* = 0.774	*r* = 0.119 *p* = 0.447	*p* = 0.082	*p* = 0.184
Intermediate (% MO subset)	*r* = 0.166 *p* = 0.286	*r* = 0.116 *p* = 0.458	*r* = 0.037 *p* = 0.814	*r* = 0.039 *p* = 0.803	***r* = 0.281** ***p* = 0.042**	*r* = 0.108 *p* = 0.491	*p* = 0.487	*p* = 0.313
Non-classical (% MO subset)	*r* = −0.110 *p* = 0.483	*r* = −0.061 *p* = 0.695	*r* = 0.239 *p* = 0.122	*r* = 0.240 *p* = 0.121	*r* = 0.126 *p* = 0.427	*r* = −0.011 *p* = 0.942	*p* = 0.164	*p* = 0.141
ACE^+^classical (% MO classical)	*r* = 0.084 *p* = 0.593	*r* = 0.047 *p* = 0.763	*r* = 0.106 *p* = 0.498	*r* = 0.110 *p* = 0.484	*r* = −0.035 *p* = 0.825	*r* = 0.034 *p* = 0.827	*p* = 0.273	*p* = 0.640
ACE^+^intermediate (% intermediate)	*r* = −0.101 *p* = 0.518	*r* = −0.112 *p* = 0.473	*r* = 0.358 *p* = 0.118	*r* = 0.301 *p* = 0.112	*r* = 0.160 *p* = 0.313	*r* = −0.105 *p* = 0.501	*p* = 0.941	*p* = 0.581
ACE^+^non-classical (% non-classical)	*r* = −0.130 *p* = 0.407	*r* = −0.115 *p* = 0.464	*r* = 0.358 *p* = 0.129	*r* = 0.359 *p* = 0.181	*r* = 0116. *p* = 0.465	*r* = −0.201 *p* = 0.196	*p* = 0.551	*p* = 0.273

The associations between individual cell subsets and vascular indices were evaluated by Spearman ranks’ correlation tests (continuous variables) or Mann-Withney U tests (categorical variables). Associations reaching statistical significance were highlighted in bold. PWV, Pulse Wave Velocity; cIMT, carotid intima-media thickness; MO, Monocytes; ACE, angiotensin converting enzyme.

**Figure 4:**
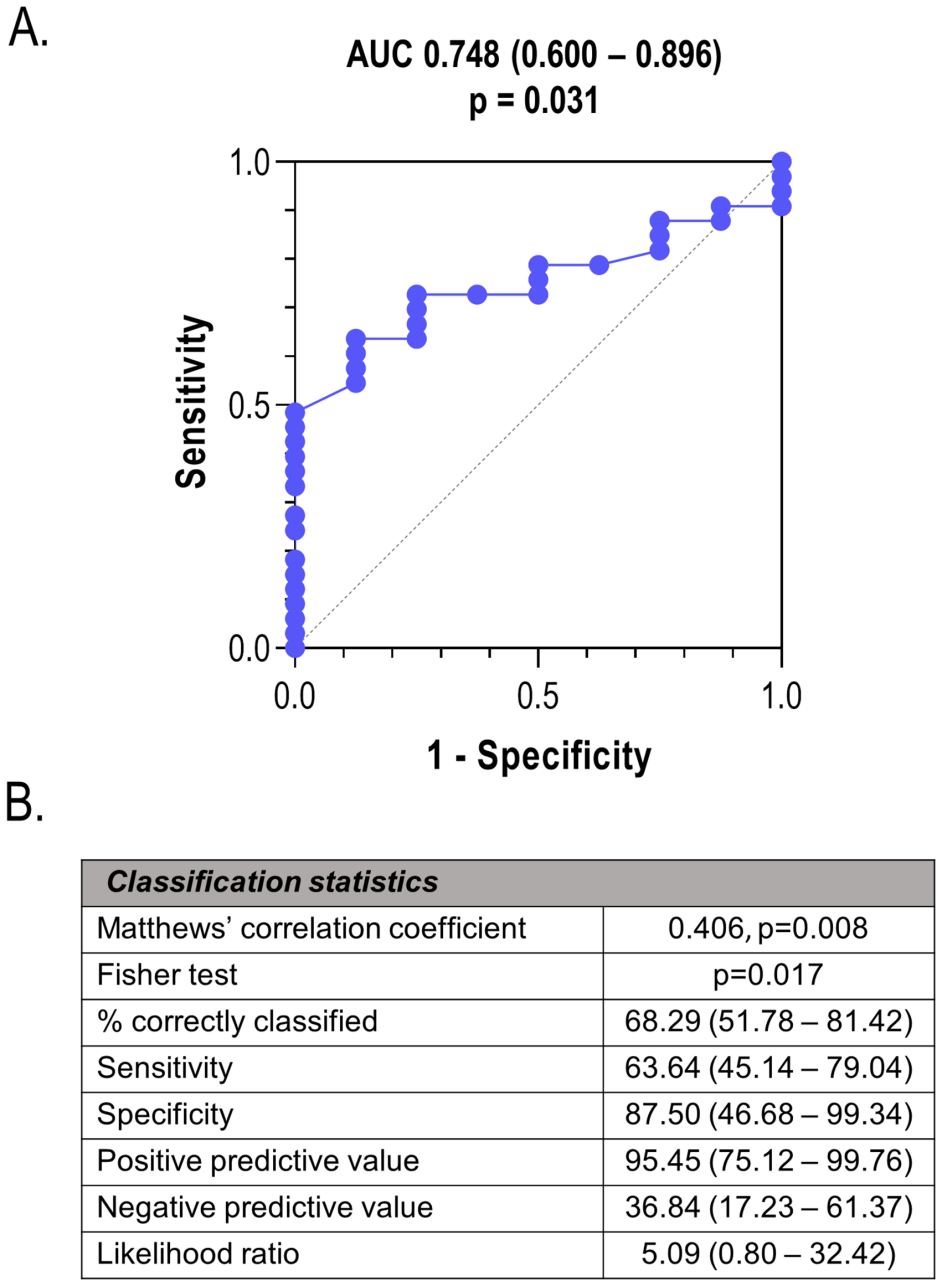
Tang levels as biomarkers of vascular stiffness. **(A)** The ability of Tang levels to discriminate between CKD patients with and without vascular stiffness (>10 m/s) was tested in a ROC curve. **(B)** Classification statistics and goodness-of-fit metrics demonstrated the validity of Tang levels (<1.63% CD3^+^, as per ROC analyses) as a biomarker.

Tang levels were found to predict PWV in univariate and multivariate models, adjusted for potential confounders (Table 3). Replacing risk factors (such as hypertension) by its surrogate measurements (such as systolic or diastolic blood pressure) did not change these results. Equivalent findings were obtained by linear regression with backward elimination after entering all the variables, hence confirming that only Tang levels (*p* = 0.002) were independent predictors. This association remained statistically significant even after adjusting for cIMT (B [95%]: −1.748 [−3.226, −0.269], *p* = 0.022), thus ruling out a confounding effect of cIMT on vascular stiffness. Of note, Tang frequency failed to independently predict cIMT in multivariate models (Supplementary Table S6), age being the main predictor of this feature. Furthermore, Tang levels showed a good discriminative power to identify patients with vascular stiffness (Figure 4A). Based on Youden and Gini indices on ROC analyses, a value of 1.63% was identified as the optimal cut-off. Classification statistics reinforced the value of Tang depletion (<1.63%) as a biomarker for vascular stiffness (Figure 4B).

**Table 3:** Analysis of Tang levels as predictors of vascular stiffness.

	Univariate models				Multivariate model			
	Beta	B	95% CI	*p*-value	Beta	B	95% CI	*p*-value
Tang, per 1%	−0.463	−2.225	−3.586, −0.864	**0.002**	**−0.403**	**−1.937**	**−3.422, −0.452**	**0.012**
Age, per 1 year	0.367	0.103	0.059, 0.247	**0.023**	0.212	0.075	−0.031, 0.182	0.101
Sex, men	−0.051	−0.285	−2.085, 1.515	0.750				
Hypertension, yes	0.254	1.769	−0.414, 3.952	0.109				
Dyslipemia, yes	−0.025	−0.157	−2.175, 1.861	0.876				
HR, per 1 unit	0.043	0.009	−0.057, 0.075	0.788				
CKD stage, per 1 unit	0.303	0.760	−0.014, 1.534	0.054	0.020	0.055	−0.816, 0.926	0.899

The associations between PWV and different potential predictors, including Tang frequency, were analyzed by linear regression in univariate analyses. Those parameters associated (*p* < 0.100) with PWV in univariate analyses were entered in a multivariate model. *p*-values reaching significance were highlighted in bold. CI, confidence interval; CKD, Chronic Kidney Disease; HR, heart rate; PWV, Pulse Wave Velocity.

All these findings revealed that immune cell populations could be related to vascular indices in the earliest stages of CKD, both at baseline and after follow-up. Tang were related to a number of vascular traits, and were confirmed to be independent predictors of vascular stiffness.

## Discussion

Vascular injury is a hallmark of the natural history of CKD. However, underlying mechanisms remain elusive. In the present study, we addressed the study of immune cell populations involved in vascular homeostasis in a cohort of non-dialysis CKD patients encompassing the whole disease spectrum. Our findings revealed profound and different alterations of these cell populations, which also showed distinct patterns in their associations with vascular indices. Importantly, these populations and their associations remained stable, hence reinforcing their potential value as biomarkers. To the best of our knowledge, this is the first study in performing such a cellular characterization of along the CKD continuum before renal replacement therapy, including early stages, as well as to provide clues on the stability of these changes in the mid-term.

A key finding from our analysis was the evaluation of immunosenescent traits within the T-cell compartment. Our data confirm that immunosenescence is a common hallmark across the whole CKD spectrum, being enhanced already in the first stages and showing no changes along progression to end-stage renal disease. These results are in line and expand previous findings from our group (11) and others (20, 21), which reported increased T-cell immunosenescence in patients under different dialysis regimens. Observing early, stage-independent, signs of immunosenescence in CKD may shed new light on its role in this condition, and it helps to understand the temporal dimension of these features. Rather than being considered as an epiphenomenon due to accumulation of exhausted cells or a late consequence of the disease process itself (22), these findings point to an initial role in disease pathogenesis. Furthermore, although immunosenescence has been related to vascular injury (23, 24), including a number of mechanisms being demonstrated *in vitro* (25), our results failed to show an association between T-cell immunosenescence and vascular subclinical endpoints.

A major breakthrough from our study was the analysis of the Tang subset. First, despite not being altered in numbers, Tang population exhibited immunosenescence traits along the whole CKD spectrum, also including the decreased CD4/CD8 ratio mirroring that of the T-cell compartment. These findings add another layer of complexity to the analysis of Tang in the field in CKD. Although senescent Tang have been described in autoimmune patients (26, 27), there was no prior evidence in CKD populations. Further analyses are needed to understand if cellular senescence could lead to a selective functional impairment in Tang. Second, Tang exhibited a progressive, stage-dependent depletion, leading to decreased numbers in the late disease stage. This finding is of special relevance as it demonstrates that Tang subset disturbances start early along the disease course, hence strengthening their potential value as a biomarker as well as therapeutic targets for preventive strategies. These notions are in line with the fact that Tang numbers were found to be associated with subclinical vascular indices, thus ruling out a potential confounding effect of clinical events to account for Tang depletion in CKD. Further analyses strengthened the performance of Tang as biomarkers of vascular stiffness. Interestingly, Tang frequency was found to be related to vascular surrogates informing altered vascular functionality (vascular stiffness) or initial vascular damage (adventitial neovascularization), rather than informing structural vascular changes, such as plaque occurrence or even vascular calcification, which represent harder endpoints. These findings align with previous studies in inflammatory chronic diseases (28, 29), thus expanding the interest on the Tang subset beyond autoimmune diseases. Taken together, these results support the role of Tang as candidate biomarkers to identify patients with altered vascular functionality but without structural arterial changes, who may benefit from a tighter management to avoid vascular progression, CV disease morbidity and thus disease burden in CKD.

Despite being unrelated to plaque occurrence, Tang levels exhibited an association with plaque phenotype. Decreased Tang in patients with calcified plaques may suggest that plaque calcification could be associated with vascular repair failure and thus, higher vascular risk. In fact, certain calcium deposition within atherosclerosis plaques has been reported to confer enhanced risk of plaque instability and thus, rupture. However, full plaque calcification has been also described to reduce plaque erosion, increase stability and thus alleviate plaque rupture odds, which challenge our previous assumption (30, 31). It is important to consider that plaque calcification results from a complex interaction and that calcification extent and plaque composition are better surrogates to inform subsequent plaque stability. Due to technical limitations, our study was not powered to assess plaque composition and hypo/hyper echogenic properties of plaques. Further studies are to be conceived to cover this gap and shed new light into the connection between circulating Tang and plaque stability, which may be also relevant beyond CKD.

Our data revealed a number of associations with PWV, which were stronger also compared with other vascular endpoints, especially in the case of Tang. This is of special relevance as PWV may be considered as a better tool to assess vascular involvement in CKD populations (3) compared to other measures such as cIMT (32). Furthermore, Tang was correlated to PWV and adventitial neovascularization in the present report, which is supported by the positive association between both measures in CKD patients in our previous report. Moreover, PWV has been found to be a predictor of clinical CV events and mortality in general population and in end-stage renal disease (16, 33, 34) and even detect aortic stiffness in earlier stages of CKD (3, 35), hence emphasizing its clinical value for patient management. Furthermore, this study showed that PWV was the only parameter of vascular damage to be correlated with both Tang cells and intermediate monocytes, strengthening the link between vascular disease and inflammation (36, 37).

Additionally, our results point to a subtle shift within the monocyte compartment toward the intermediate subset, which were associated with vascular stiffness although to a lower extent. These findings are in line with previous evidence from our group (11), and reinforce the hypothesis that monocyte alterations may be a late event along CKD continuum linked to CKD pathogenesis itself rather than directly involved on vascular injury, at least in subclinical outcomes. We have previously proposed that this may be related to the Th1-skewed response observed in CKD (38, 39). Immunosenescence has been related to aberrant Th1 responses in several scenarios (19, 40), and T-cell immunosenescence was herein observed to occur early within CKD natural history. Therefore, it is tempting to speculate that monocyte changes in CKD may be a consequence of an early T-cell exhaustion, Th1 shift being the missing link in this scenario. In fact, previous results from our group have reported a connection between IFNg serum levels and monocyte heterogeneity in CKD patients (11). Of note, it has also been proposed that CD16^+^ monocytes can be reflective of cellular senescence (41), hence closing the gap between monocyte compartment and immunosenescence. Furthermore, our results failed to show differences in ACE expression within the monocyte compartment, opposed to what was found in our previous cohort of patients undergoing dialysis (11, 42). It is tempting to speculate that in early CKD stages, ACE expression may be still regulated by the renin-angiotensin-aldosterone system, whereas this regulatory loop is abrogated in end-stage renal disease. Lack of differences in vitamin D levels between patients and control populations may support this notion. However, it is unclear if ACE expression on immune cells is entirely under this canonical regulation. Actually, ACE expression on monocytes was found to be unrelated to traditional risk factors, anti-hypertensive medications (including ACE inhibitors) and serum ACE activity (42), and differences between lymphoid and non-lymphoid cells have been also reported (43). In fact, previous results suggest a link between ACE expression on monocytes and IFNg levels (11), hence underlining the connection between monocyte alterations and immunosenescence pathways. Taken together, these pieces of evidence further support the hypothesis that immunosenescence may play a more important role in CKD pathogenesis, through monocyte polarization, than in vascular indices themselves, thereby ruling out a major role as biomarkers.

Our analysis has revealed that differences in all immune cell population between CKD patients and control individuals were maintained after an 18-months follow-up, and so did their associations with vascular endpoints. This aligns with the fact that the latter experienced almost no changes (with the exception of plaque burden) along this period (3), and clinical progression of CKD stages was limited. Therefore, immune cell populations can be considered stable, at least in the absence of clinical progression or major changes in therapeutic management. This builds upon previous evidence on 6-month follow-ups on myeloid populations performed by our group (44), thus expanding the timeframes. Taking into account the chronic dimension of CKD, these findings provide another advantage which favors the validity of the cell populations analyzed here as robust biomarkers. Based on these lines of evidence, larger longitudinal trials are warranted to evaluate their potential added value for additional (and harder) clinical endpoints.

The main limitation of this work lies in the reduced number of patients on each CKD stage, so further studies are needed to replicate these findings in middle-aged individuals with CKD for clinical validation, as well as with longer follow-ups to evaluate associations with harder clinical endpoints. In the future it would be interesting to design experimental approaches to understand the mechanistic role of immunosenescent and Tang cells on the vasculature in the CKD setting. However, this study presents several strengths: it involves early, real-world CKD patients, it is based on a comprehensive set of subclinical vascular indices, and it demonstrates the applicability of Tang as an interesting biomarker of vascular impairment in pre-dialysis CKD patients, while reinforcing the use of non-invasive techniques, such as PWV.

In conclusion, the results herein presented support that pre-dialysis CKD stages are hallmarked by alterations in immune cell populations related to vascular homeostasis, including early T-cell immunosenescence traits and a stage-dependent Tang depletion, which was independently related to vascular stiffness. All these features were replicated upon follow-up, thus providing validation toward our results. Remarkably, our findings pave the ground for future studies addressing the functional contribution of these cellular mediators at the local level, as well as assessing their potential predictive value in the long-term and their potential modulation as disease targets for preventive strategies in the clinical setting.

## Data Availability

The raw data supporting the conclusions of this article will be made available by the authors, without undue reservation.
