# Supplementary material for: Novel T-cell subsets as non-invasive biomarkers of vascular damage along the predialysis stages of chronic kidney disease
Source: Front Med (Lausanne). 2024 Dec 9;11:1460021. doi: 10.3389/fmed.2024.1460021 (PMC11663642; doi:10.3389/fmed.2024.1460021)

SUPPLEMENTARY MATERIALS

Supplementary Table 1: Demographic, laboratory and clinical parameters of study participants at the 18-month follow-up. Variables were summarized as mean±SD, median [interquartile range] or n (%). Differences were assessed by chi-square tests, Student t tests or Mann-Withney U tests, as appropriate.

	**HC** (n=37)	**CKD** (n=35)	**p-value**
Demographics			
Age (years)	68.0±4.8	68.7±8.4	0.651
Sex (women/men)	20/18	18/25	0.456
Clinical features			
BMI (kg/m^2)	27.2±4.6	28.2±4.4	0.318
Systolic blood pressure (mm Hg)	128 [14]	136 [17]	0.024
Diastolic blood pressure (mm Hg)	73.0 [15.0]	74.0 [14.0]	0.978
Heart rate (beats per min)	62±9	69±16	0.026
eGFR (mL/min/1.73 m^2)	83.0 [10]	29.0 [21.5]	<0.001
Creatinine (mg/dL)	0.81 [0.20]	2.09 [1.47]	<0.001
Calcium (mg/dL)	9.53±0.32	9.66±0.43	0.154
Phosphorus (mg/dL)	3.66 [0.49]	3.60 [0.49]	0.955
PTH (pg/mL)	51.0 [21.0]	94.0 [79.5]	<0.001
FGF23 (pg/mL)	59.40 [34.04]	160.90 [208.55]	<0.001
Calcidiol (ng/mL)	28.80±12.23	30.87±14.07	0.508
Traditional risk factors, n (%)			
Hypertension	16.2	77.1	<0.001
Dyslipemia	27.0	64.9	<0.001
Treatments, n (%)			
Anti-hypertensive drugs (any)	18.9	77.1	<0.001
ACEi	17.1	54.3	0.003
Diuretics	11.4	42.9	0.007
Statins	21.6	88.6	<0.001
Paracalcitol	2.9	25.7	0.017
Calcimimetics	0	5.7	0.473

HC, healthy controls; CKD, chronic kidney disease; BMI, body mass index; eGFR, estimated glomerular filtration rate; PTH, parathyroid hormone; FGF23, fibroblast growth factor 23; ACEi, angiotensin converting enzyme inhibitors.

Supplementary Table 2: Effect of traditional risk factors and medications on T-cell subsets. Differences were measured by Mann-Whitney U tests, and p-values for each comparison are shown.

	**CD4+CD28null** (% CD4+)	**Tang** (% CD3+)
Traditional risk factors		
Hypertension	p=0.471	p=0.818
Dyslipemia	p=0.430	p=0.820
Treatments		
Anti-hypertensive drugs (any)	p=0.118	p=0.295
ACEi	p=0.679	p=0.855
Diuretics	p=0.922	p=0.237
Statins	p=0.939	p=0.571
Anti-diabetics	p=0.222	p=0.302
Paracalcitol	p=0.965	p=0.965
Calcimimetics	p=0.886	p=0.866

ACEi, angiotensin converting enzyme inhibitors.

Supplementary Table 3: Effect of traditional risk factors and medications on monocyte subsets and ACE expression. Differences were measured by Mann-Withney U tests, and p-values for each comparison are shown.

	**Classical** (% MO gate)	**Intermediate** (% MO gate)	**Non-classical** (% MO gate)	**ACE^{+} classical** (% classical)	**ACE^{+} intermediate** (% intermediate)	**ACE^{+} non-classical** (% non-classical)
Traditional risk factors						
Hypertension	p=0.635	p=0.866	p=0.795	p=0.571	p=0.550	p=0.398
Dyslipemia	p=0.183	p=0.640	p=0.800	p=0.679	p=0.277	p=0.314
Treatments						
Anti-hypertensive drugs (any)	p=0.281	p=0.453	p=0.105	p=0.826	p=0.567	p=0.168
ACEi	p=0.865	p=0.050	p=0.990	p=0.262	p=0.113	p=0.197
Diuretics	p=0.588	p=0.133	p=0.112	p=0.112	p=0.104	p=0.279
Statins	p=0.416	p=0.725	p=0.988	p=0.550	p=0.550	p=0.433
Anti-diabetics	p=0.452	p=0.818	p=0.095	p=0.288	p=0.302	p=0.433
Paracalcitol	p=0.588	p=0.133	p=0.942	p=0.112	p=0.104	p=0.281
Calcimimetics	p=0.199	p=0.244	p=0.434	p=0.718	p=0.066	p=0.268

MO, monocytes; ACE, angiotensin converting enzyme; ACEi, ACE inhibitors.

Supplementary Table 4: Analysis of changes in immune cell populations according to CKD progression status. Patients were classified according to their progression status at 18 months and differences in immune cell subsets were measured by Mann-Withney U tests. Variables were summarized as median (interquartile range).

	**CKD progression (18 months)**		**p-value**
	**No** (n=25)	**Yes** (n=10)	
T-cell subsets			
CD4+CD28null (% CD4+)	26.39 (7.29)	29.80 (8.20)	0.287
CD4+ (% CD3+)	56.59 (21.83)	58.40 (33.12)	0.928
CD8+ (% CD3+)	35.95 (21.13)	36.07 (26.44)	0.872
Tang (% CD3+)	1.44 (1.00)	1.96 (0.86)	0.186
TangCD28null (% Tang)	51.61 (12.41)	51.75 (13.28)	0.900
Monocyte subsets			
Classical (% MO subset)	69.88 (12.30)	71.54 (9.72)	0.999
Intermediate (% MO subset)	5.10 (2.75)	6.02 (2.93)	0.240
Non-classical (% MO subset)	16.51 (8.61)	15.30 (5.28)	0.900
ACE+classical (% MO classical)	1.59 (2.21)	0.60 (1.10)	0.113
ACE+intermediate (% intermediate)	8.93 (7.31)	5.42 (7.04)	0.577
ACE+non-classical (% non-classical)	18.91 (13.11)	19.13 (12.86)	0.760

CKD, Chronic Kidney Disease; MO, monocytes; ACE, angiotensin converting enzyme.

Supplementary Table 5: Analysis of the associations between immune cell subsets and subclinical vascular indices after 18-months follow-up. The associations between individual cell subsets and vascular indices were evaluated by Spearman ranks' correlation tests (continuous variables) or Mann-Whitney U tests (categorical variables). Associations reaching statistical significance were highlighted in bold.

	**Number of carotid neovasa**	**Area of carotid neovasa (mm^2/mm %)**	**Number of femoral neovasa**	**Area of femoral neovasa (mm^2/mm %)**	**PWV (m/s)**	**cIMT (mm)**	**Carotid plaque (Yes/No)**	**Femoral plaque (Yes/No)**	**Kauppila index**
T-cell subsets									
Tang (% CD3+)	**r=-0.368 p=0.032**	**r=-0.401 p=0.019**	r=-0.127 p=0.474	r=-0.127 p=0.414	**r=-0.341 p=0.048**	**r=-0.364 p=0.034**	p=0.800	p=0.741	r=-0.184 p=0.306
TangCD28null (%Tang)	r=-0.114 p=0.468	r=-0.078 p=0.618	r=-0.256 p=0.097	r=-0.259 p=0.093	r=-0.029 p=0.859	r=-0.047 p=0.763	p=0.339	p=0.778	
CD4+CD28null (% CD4+)	r=-0.172 p=0.331	r=-0.122 p=0.492	r=0.233 p=0.185	r=0.233 p=0.184	r=0.104 p=0.559	r=-0.165 p=0.351	p=0.744	p=0.341	r=0.251 p=0.160
Monocyte subsets									
Classical (% MO subset)	r=0.106 p=0.552	r=0.093 p=0.599	r=-0.193 p=0.274	r=-0.198 p=0.262	r=-0.013 p=0.944	r=0.028 p=0.875	p=0.344	p=0.145	r=0.288 p=0.104
Intermediate (% MO subset)	r=-0.189 p=0.284	r=-0.186 p=0.294	r=0.151 p=0.394	r=0.157 p=0.374	**r=0.383 p=0.026**	r=-0.040 p=0.822	p=0.143	p=0.439	r=-0.227 p=0.204
Non-classical (% MO subset)	r=-0.143 p=0.420	r=-0.167 p=0.344	r=0.194 p=0.272	r=0.200 p=0.258	r=0.090 p=0.612	r=-0.062 p=0.729	p=0.856	p=0.110	r=-0.306 p=0.083
ACE+classical (% MO classical)	r=0.326 p=0.059	r=0.282 p=0.107	r=0.181 p=0.306	r=0.185 p=0.294	r=-0.094 p=0.599	r=0.151 p=0.394	p=0.143	p=0.775	r=-0.263 p=0.139
ACE+intermediate (% intermediate)	r=-0.259 p=0.138	r=-0.301 p=0.084	r=0.318 p=0.064	r=0.381 p=0.054	r=0.026 p=0.883	r=-0.299 p=0.086	p=0.403	p=0.416	r=0.029 p=0.874
ACE+non-classical (% non-classical)	r=0.249 p=0.156	r=0.166 p=0.348	r=0.009 p=0.958	r=0.008 p=0.962	r=-0.155 p=0.381	r=0.139 p=0.432	p=0.885	p=0.466	r=-0.097 p=0.590

PWV, Pulse Wave Velocity; cIMT, carotid intima-media thickness; MO, Monocytes; ACE, angiotensin converting enzyme.

Supplementary Table 6: Analysis of Tang levels as predictors of cIMT. The associations between cIMT and different potential predictors, including Tang frequency, and PWV were analyzed by linear regression in univariate analyses. Those parameters associated with cIMT in univariate analyses were entered in a multivariate model. p-values reaching significance were highlighted in bold.

	Univariate models				***Multivariate model***			
	**Beta**	**B**	**95% CI**	**p-value**	**Beta**	**B**	**95% CI**	**p-value**
Tang, per 1%	-0.463	-2.225	-3.586, -0.864	**0.002**	-0.179	-0.049	-0.131, 0.033	0.235
Age, per 1 year	0.482	0.010	0.004, 0.016	**<0.001**	**0.401**	**0.008**	**0.002, 0.014**	**0.008**
Sex, men	0.082	0.029	-0.082, 0.140	0.600				
Hypertension, yes	0.137	0.062	-0.079, 0.202	0.887				
Dyslipemia, yes	-0.080	-0.031	-0.153, 0.091	0.610				
HR, per 1 unit	-0.133	-0.002	-0.006, 0.003	0.401				
CKD stage, per 1 unit	0.302	0.049	0.001, 0.097	**0.049**	0.112	0.018	-0.031, 0.067	0.460

Supplementary Figure 1: Analysis of Tang levels according to plaque calcification status. Differences in Tang levels according to plaque calcification status in CKD patients were evaluated by Mann-Withney U tests at baseline (g=0.51) (A) and after follow-up (B). Scatter plots depict the distribution of individual values (each dot corresponds to one individual). Upper, medium and lower bars represent 75th, 50th (median) and 25th percentiles.

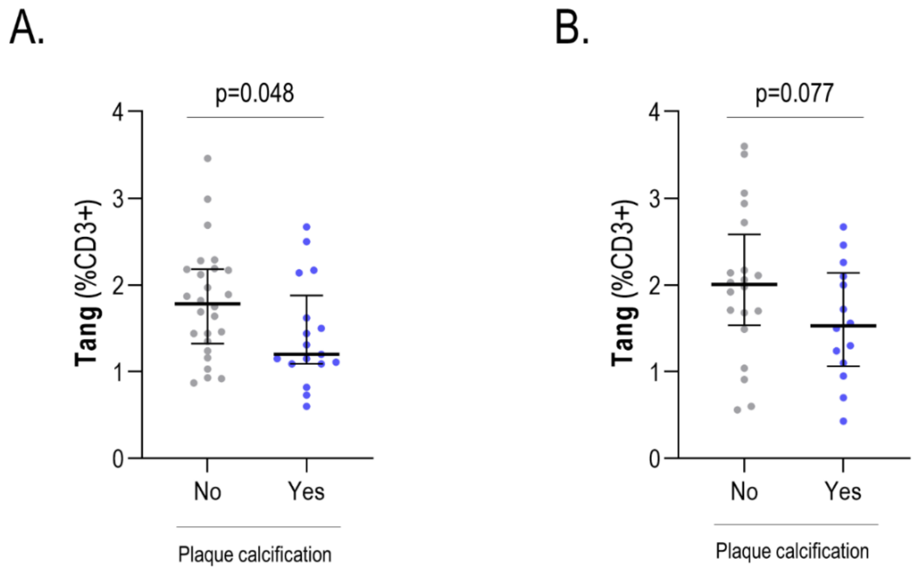

Supplement: Supplementary file 1 [file Data_Sheet_1.pdf]
